# p27^kip1^: a target for tumor therapies?

**DOI:** 10.1186/1747-1028-2-13

**Published:** 2007-05-09

**Authors:** Irina Nickeleit, Steffen Zender, Uta Kossatz, Nisar P Malek

**Affiliations:** 1 Institute for Molecular Biology, Hannover Medical School, Hannover, Germany; 2 Dept. of Gastroenterology, Hepatology and Endocrinology, Hannover Medical School, Hannover, Germany

## Abstract

The cyclin kinase inhibitor p27^kip1 ^acts as a potent tumor supressor protein in a variety of human cancers. Its expression levels correlate closely with the overall prognosis of the affected patient and often predict the outcome to different treatment modalities. In contrast to other tumor suppressor proteins p27 expression levels in tumor cells are frequently regulated by ubiquitin dependent proteolysis. Re-expression of p27 in cancer cells therefore does not require gene therapy but can be achieved by interfering with the protein turnover machinery. In this review we will summarize experimental results which highlight the potential use of p27 as a target for oncological therapies.

## Review

### Introduction

Oncology, the science which aims to understand the development, diagnosis, treatment, and prevention of human cancers, has undergone major changes in recent years. The introduction of targeted drugs which interfere with signal transduction cascades to block tumor cell proliferation is only the beginning of a fundamental change in the way cancer is treated. These significant advances in clinical oncology were made possible through the detailed analysis of the molecular origins of human cancers [1]. From these studies we learned that most human cancers undergo genetic changes to escape external control mechanisms which normally regulate cell division. Cell divison in turn is regulated by a highly conserved group of proteins which together constitute the basic cell divison machinery that controls the cell cycle [2]. Changes in the expression or activity levels of these proteins are almost always detected in human cancer cells and in many cases are targets of genetic alterations which lead to cancer formation. The cyclin kinase inhibitor p27^kip1 ^plays a central role in the suppression of tumorigenesis in a variety of human cancers [3]. The best understood function of this protein is the inhibition of the activity of cyclin E or A containing cdk2 (cyclin dependent kinase 2) complexes. This function enables p27 to regulate the progression of a cell from a quiescent state into the G1 phase and from the G1 phase into S-phase. Several external signals either induce an increase (TGFβ, serum starvation, contact inhibition and others) or a decrease (serum stimulation, estrogen, Il2, PDGF and others) in p27 cellular levels thereby allowing p27 to become a central mediator of mitogenic and anti-mitogenic pathways [4]. p27 expression levels are regulated by transcriptional, translational and post-translational mechanisms. Of particular importance for the development of human cancers is the ubiquitin dependent degradation of p27 by the proteasome. This pathway is controlled by phosphorylation of p27 at a conserved Threonine (T187) by cyclin E/cdk2 complexes and by the expression of the F-Box protein skp2 which facilitates polyubiquitylation of p27 by the SCF complex [5-7].

In addition to being expressed at insufficient levels p27 can also be functionally inactivated by being mislocalized to the cytoplasm or through phosphorylation events which prevent binding to its critical cellular targets i.e cyclin E and A/cdk2 complexes. The discovery that phosphorylation by AKT and SRC kinases at either T157 (AKT) or Tyrosine 74, 88 (SRC) induces cellular mislocalization or functional inactivation and degradation has broadened the spectrum of oncogenic mechanisms which disable p27, i.e., as a tumor suppressor protein [8,9].

The fact that p27 expression levels and its cellular functions are under the control of well characterized post-translational mechanisms makes it an interesting target for pharmaceutical interventions. Such therapies would aim at the stabilization of p27 in tumor tissues by interfering with the enzymatic machinery that controls p27 destruction. Given the enormous efforts and costs connected to the development of such new drugs proper selection of potential drug targets is of major importance. In this review we will ask if the cyclin kinase inhibitor p27^kip1 ^could be a valuable target for pharmaceutical intervention.

## Frequency of deregulation of p27 in human cancer

An overwhelming number of studies have shown that reduced expression levels of p27 in primary cancer tissue correlates with reduced overall and progression free survival as well as poor response to chemotherapies or targeted treatments. Inverse correlations between patient prognosis and p27 levels were shown in breast, prostate, bladder, lung, glia, liver, larynx, ovary, stomach, and other tissues. This concept has been summarized extensively in several excellent reviews [3,4,10]. While certain tumor types for example AML and ALL show larger deletions of the genomic area in which p27 is located no somatic mutations of the remaining allele have been identified [11]. In general and in strong contrast to other tumor suppressor proteins somatic mutations in the p27 locus are very rare in human cancers. A noteable exception from this rule is the recent finding that a rat strain which displays a MEN (multiple endocrine neoplasia) like phenotype syndrome (MENX) shows a mutation in the rat p27 locus which leads to a reduction of p27 levels in different rat tissues. Interestingly the authors also identify a human patient with a MEN like syndrome with a germline mutation in the p27 locus. Nevertheless this study is a rare example of a genetic alteration of the p27 locus [12].

The majority of studies in which the expression levels of p27 were measured in human tumor specimens conclude that the reduction of p27 levels is caused by an increase in protein degradation. Since the discovery of skp2 as the essential F-Box protein that controls p27 stability several examples of an inverse correlation between skp2 and p27 expression in different tumors have been published [13]. Some studies even showed that tissues from tumors which express low levels of p27, i.e, colon carcinoma, mantle cell lymphoma, small cell lung cancer and others, also displayed an increase in p27 degradatory activity [14,15].

## Validation of p27 contribution to tumorigenesis in model systems

This correlative data from human cancer samples in which the number of p27 expressing cells was determined by immune staining is now supported by a great number of mouse studies. Loss of p27 in the mouse confers only a relatively mild tumor phenotype leading to the development of pituitary adenomas and prostate hyperplasia with increasing age [16-18]. However Fero and co-workers showed that p27 knockout mice have a greatly increased susceptibility to the development of cancers after treatment with chemical carcinogens or irradiation [19]. Their study also showed that loss of just one p27 allele is sufficient to increase the overall number of tumors that arise and shortens the overall survival of the affected mouse. These initial studies were followed by a great number of experiments in which the contribution of p27 loss to the development of various cancer types in mouse models has been evaluated. For example, loss of p27 on a heterozygous PTEN background leads to a dramatic increase in the incidence of precursor lesions which progress to carcinoma in situ or even invasive carcinomas of the prostate, while PTEN mice with intact p27 expression develop fewer precursor lesions which do not progress to more malignant states [20]. Similar results were found in models for pituitary cancer, lymphoma, testicular cancer and others [21,22]. In colon cancer models loss of p27 cooperated with mutation in the APC (min) gene but showed no cooperativity with mutations in the Smad3 gene which is part of the TGFβ pathway [23]. In general these studies highlight the fact that a reduction of p27 levels promotes tumor development fueled by most but not all oncogenic events.

## Interference with p27 activity reverses or blocks the malignant phenotype

The observations made in mouse models in combination with the facts that p27 is primarily (but not exclusively) regulated by post-translational mechanisms has spurred interest in developing methods which target the enzymatic machinery that regulates the degradation of p27. Studies in which p27 was over-expressed through different types of transfection or infection techniques had shown that re-expression of p27 in tumor cells (brain, lung, breast) often induces apoptosis [24,25]. The mechanism by which p27 overexpression in tumor cells induces apoptosis is largely unknown. Overexpression of p27 in primary non-transformed cells however usually arrests the cell in G0/G1 thereby providing some degree of specificity to a "p27 therapy". Based on the detailed analysis of p27 degradation by the skp2 dependent SCF complex it seems now possible to interfere with p27 degradation in a much more specific way. One way is the direct interference with skp2 expression either by siRNA or blocking antibodies. Both treatments were shown to reduce the ability of skp2 to degrade p27 thereby blocking cell proliferation of lung cancer, oral cancer and melanoma cells. [26-28]

By using a mouse model in which expression of a degradation resistant version of p27 (p27T187A) led to a reduction in the number of adenomatous polyps which progressed to invasive intestinal carcinomas, we recently demonstrated that p27 stabilizing treatments might be of clinical value. This study showed that in contrast to wildtype p27, p27T187A expression was maintained throughout the course of intestinal carcinogenesis and correlated with the delayed progression of the intestial tumors in the p27T187A mouse. Because the expression of the degradation resistant version of p27 is under the control of the genomic p27 promoter the cellular levels of the T187A form that were reached in this study were exclusively caused by a reduction in the rate of protein turnover. This study therefore showed that treatments aimed at reducing p27 degradation might be of therapeutic value in patients with colon cancer. Interestingly the stabilization of p27 did not lead to a reduction in the proliferation rates of the tumor tissue but instead affected the de-differentiation of the pre-cancerous adenoma into malignant carcinomas [29]. Similar observations have previously been made in human colon carcinomas in which p27 expressing tumors showed a more differentiated phenotype than low expressing cancers [30]. These studies point to the interesting question as to how p27 expression might interfere with cellular differentiation programs. Indeed, studies in colon epithelial cells showed that while the overexpression of both p16 and p27 led to a reduction in cellular proliferation rates the expression of only p27 induced differentiation associated changes in morphology as well [31]. Strong evidence for a role of p27 in differentiation programs comes from studies of xenopus neurogenesis. Here overexpression of a non cdk inhibitory mutant of p27^xic1^, the xenopus homologue of p27^kip1^, induces differentiation of immature neurons [32]. This observation was recently validated in a knock-in mouse model in which p27^c-k- ^(a mutant form of p27 which cannot bind cyclin or cdk) promoted normal brain development while p27 KO mice show specific defects in this process [33]. In analogy to the results in xenopus neural development p27 seems to stabilize the protein neurogenin which is essential for neural differentiation by preventing its degradation. It is therefore conceivable that drugs which prevent p27 degradation will in addition to causing apoptosis affect tumor cell de-differentiation.

## Potential risks of p27 stabilizing therapies- the dark side of p27

Based on our knowledge regarding the function and regulation of p27 in normal and tumor cells it appears that a p27 stabilizing therapy could be clinically beneficial. However the fact that the p27 gene is only very rarely mutated in human cancers also points to potential risks associated with such an approach. Intuitively one would argue that if a tumor cell does not mutate a tumor suppressor gene this could indicate that the protein might have a tumor promoting function at least in the early stages of tumor initiation. If such functions would exist stabilization of p27 in tumor cells might actually not inhibit tumorigenesis but promote it. In fact several tumor promoting functions of p27 are conceivable.

First, p27 as well as p21 can function as assembly factors for cyclin D/cdk4 complexes thereby promoting Rb phosphorylation and cell cycle progression [34]. It is however difficult to experimentally determine to which extent the assembly function of p27 contributes to tumorigenesis. Loss of p27 should impair cyclin D/cdk4 activation (at least when p21 is deleted at the same time), however some p27 KO cells and tissues show increased levels of cdk2 dependent kinase activity thereby essentially circumventing the need for cyclin D kinase activation. Nevertheless in an ErbB2 dependent mouse breast cancer model Muraoka and co-workers showed that while loss of *one *p27 allele accelerated breast cancer development loss of *both *alleles decreased cyclin D dependent kinase activity which correlated with an increased tumor latency [35]. A similar effect was shown in mice in which the combined loss of the homeobox gene Nkx3.1 and p27^kip1 ^on a PTEN heterozygous background showed a reduced tumor incidence as compared to p27 heterozygous, Nkx3.1-/-, PTEN +/- mice [36]. In this model heterozygous p27 mice showed a significantly higher expression of cylin D than p27 knockout mice. Also heterozygous p27 knockout mice are more prone to urtehane induced lung tumorigenesis then p27 knockout mice [37]. These studies indicate that in different tumor models some p27 expression is necessary to allow tumor-initiation in breast, lung and prostate tissue. A p27 stabilizing drug would therefore need to induce p27 expression close to wildtype levels to be of clinical benefit. Otherwise re-expression of p27 in tumors could facilitate the expression and activation of cyclin D kinase activity thereby promoting tumor progression.

Studying phosphorylation dependent regulation of p27 we recently identified Threonine 198 in p27 as a phosphorylation site which controls binding of p27 to cyclin D/cdk4 complexes. Phosphorylation at T198 is required to form cyclin D/cdk4 complexes which in turn phosphorylate Rb to allow the activation of an E2F dependent transcriptional program. Interestingly AKT kinase has been shown to phosphorylate p27 at T198 while being itself an E2F target gene. Expression of p27 in early G1 might therefore help to induce AKT expression which in turn promotes cyclin D/cdk4 assembly through phosphorylation of p27 at T198. In this scenario p27 would be part of a positive feedback loop which promotes progression through this early part of the G1 phase. Expression of a non-phosphorylatable version of p27 (p27T198A) interferes with timely Rb phosphorylation thereby blocking cell cycle progression [38]. The cellular response to p27 overexpression might therefore be regulated by the activity of other signalling cascades i.e. AKT activity which will also impinge on the ability of a p27 stabilizing drug to block tumor cell proliferation.

In addition to its function in cell cycle control other non-cdk inhibitory functions of p27 have been identified in recent years. These functions would also be affected by treatments which lead to an increase in the cellular levels of p27. Particularly interesting was the finding that p27 is involved in the regulation of cell migration. In an important paper, Besson and Roberts showed that p27 knockout cells have a significantly reduced ability to migrate due to a defect in down regulating RhoA activity [39]. The ability of p27 to regulate RhoA was indeed independent of its function as a cyclin kinase inhibitor as a mutant version of p27^c-k- ^also induced migration in p27 knockout cells. Essentially and as discussed by the authors this observation suggests that loss of p27 should impair the ability of a tumor cell to migrate thereby reducing its capacity to invade the surrounding tissue or metastasize to other organs. Recent data suggests that some cancers metastasize relatively early in the course of their development; breast cancer is a prominent example of such an early metastasizing tumor [40]. However breast cancers also show a very strong correlation between low p27 levels and reduced prognosis of the affected patients [41]. Maybe early metastasis formation is promoted in tumor cells which still express sufficient levels of p27 to suppress RhoA activity while the degradation of p27 allows the rapid proliferation of the daughter cells.

Breast cancers also show an additional feature of p27 inactivation namely the exclusion of p27 from the nucleus. Work by several laboratories convincingly showed that phosphorylation of p27 by AKT at T157 and also at T198 is required for nucleo-cytoplasmic transport [42-46]. Cytoplasmic expression of p27 in breast cancer cells and in Barretts associated adenocarcinoma of the esophagus correlated with a reduced prognosis, indicating that cytoplasmic localization of p27 inactivates the tumor suppressor function of the protein [47]. However these observations do not exclude the possibility that cytoplasmic p27 gains additional functions which might even favour tumor cell proliferation or survival. A recent study by Wu and Arteaga addressed this problem experimentally by expressing a mutant form of p27 which cannot localize to the nucleus in MCF7 breast cancer cells. In cells in which p27 is normally cytoplasmic loss of p27 destabilized AKT while overexpression lead to an increase in AKT stability and tumor cell survival. The authors then extended their study to a series of human breast cancer specimens in which they showed that cytosolic p27 expression levels correlated with the expression levels of AKT and to a lesser degree the activiation of AKT as measured by phospho-AKT staining [48]. This data therefore points to an anti-apoptotic function of cytosolic p27 mediated at least in part through the activation of AKT kinase. Further support for an anti-apoptotic function of p27 was also shown in leukemic cells in which p27 can confer resistance to apoptosis after treatment with chemotherapeutic drugs. These experiments suggested that p27 itself can be cleaved by caspases to yield smaller fragments which protected the cell against apoptosis while non-cleavable forms of p27 do not [49]. Moreover p27 conferred chemotherapy resistance to tumor cells grown as spheroids most likely through the induction of a G1 arrest while reduction of p27 expression allowed efficient killing of the respective cells. p27 stabilization could therefore affect the efficiency of chemotherapeutic drugs by limiting the number of cycling cells *and *reduce the sensitivity against apoptosis in certain cell types [50].

As discussed before, the skp2/p27 interaction represents an attractive target for pharmacological intervention. However loss of skp2 leads to severe alterations in cell physiology, specifically centrosome overamplification and DNA re-replication cycles which lead to largely increased chromosome numbers in skp2 knockout hepatocytes and other cell types [51,52]. It is unclear whether the acute loss of skp2 function would induce the same changes at the organismal level or would primarily increase the expression of p27. Tumor studies in skp2 knockout mice and specifically in skp2 heterozygous mice could shed light on these questions.

## Conclusion

The strong correlation between p27 levels and patient survival in a variety of human malignancies underscores the important contribution of p27 to human carcinogenesis. These observations were experimentally proven in numerous mouse studies in which loss or reduction of p27 expression synergized with different oncogenes. Given the importance of ubiquitin dependent degradation mechanisms for the turnover of p27, therapies aimed at the stabilization of p27 could therefore be of clinical value. Nevertheless recent studies revealed additional functions of p27 specifically the regulation of cell migration and the interaction with signal transduction cascades which point to potential risks connected with the stabilization of p27. Through use of the existing mouse models which express mutant forms of p27 it should however be possible to identify those types of cancer in which p27 stabilization is clinically beneficial. One such example is intestinal cancer in which the results obtained by studying human colon carcinoma samples match the observations made in mouse lines which express more stable forms of p27, namely that preventing p27 turnover represents an interesting new target for molecular cancer therapies.
